# Complete Genome Sequence of *Bacillus subtilis* Strain PY79

**DOI:** 10.1128/genomeA.01085-13

**Published:** 2013-12-19

**Authors:** Jeremy W. Schroeder, Lyle A. Simmons

**Affiliations:** Department of Molecular, Cellular, and Developmental Biology, University of Michigan, Ann Arbor, Michigan, USA

## Abstract

*Bacillus subtilis* is a Gram-positive soil-dwelling and endospore-forming bacterium in the phylum *Firmicutes*. *B. subtilis* strain PY79 is a prototrophic laboratory strain that has been highly used for studying a wide variety of cellular pathways. Here, we announce the complete whole-genome sequence of *B. subtilis* PY79.

## GENOME ANNOUNCEMENT

*Bacillus subtilis* has been studied under laboratory conditions for >100 years, yielding tremendous insight into the biology of Gram-positive bacteria. Laboratory studies have primarily used the strains *B. subtilis* PY79 and JH642 (1). JH642 is auxotrophic and contains a number of phage and integrative conjugative elements (2–4). PY79 is a prototroph lacking many of the mobile genetic elements studied in JH642 (4, 5). The whole-genome shotgun sequence for JH642 is available, but it contains 286 nucleotides located in regions of ambiguous sequence. The JH642 genome sequence and those of other *B. subtilis* strains facilitate in-depth studies of biological mechanisms (3, 6). It is surprising, then, that although PY79 has been one of the most widely used laboratory strains, its genome sequence has been unavailable. Here, we report the complete genome sequence of *B. subtilis* PY79, generated using two sequencing platforms, PacBio RS II and HiSeq 2000 (Illumina). The PY79 genome is 154,156 nucleotides shorter than that of JH642, and, using the script run-mummer3, we found there are 3,641 single-nucleotide polymorphisms (SNPs) between JH642 and PY79 (7). Our results provide the first publicly available complete reference genome for this highly studied *B. subtilis* strain.

PY79 genomic DNA was isolated by phenol-chloroform extraction (8), and a 15-kb insert library was prepared for sequencing using two single-molecule real-time (SMRT) cells on a Pacific Biosciences RS II sequencer. The resulting mean subread length was 3.57 kb. The HGAP protocol implemented in smrtanalysis version 2.0.1 was used to assemble the PY79 genome (9). This resulted in two contigs in the HGAP output, the first of which is short, at exactly 13,000 bases in length, with 5.3× mean coverage. The second contig is 4,060,232 bases long, with 156× mean coverage. Because of its short length and low coverage, we eliminated the first contig from further analysis. The long contig was circularized, and overlaps from the ends were removed using the minimus2 script in the AMOS package (10). The circularized genome was then used as a reference for realignment twice using our original PacBio data and additionally corrected by realignment with high-coverage (464×) 50-base paired-end reads from the HiSeq 2000 platform. This correction step resulted in a complete *B. subtilis* PY79 chromosome sequence that is 4,033,459 bases long.

Genes were predicted using the RAST server (11). RAST located 4,278 features, including 4,140 coding sequences, 30 rRNA genes, and 86 tRNA genes.

### Nucleotide sequence accession number.

The whole-genome sequence of PY79 is available from DDBJ/EMBL/GenBank databases with accession no. CP006881. PY79 and many derivatives are available from the Bacillus Genetic Stock Center (http://www.bgsc.org/).
